# Late Follicular Phase Ovarian Stimulation Without Exogenous Pituitary Modulators

**DOI:** 10.3389/fendo.2020.00487

**Published:** 2020-08-13

**Authors:** Xiuxian Zhu, Jing Ye, Yonglun Fu

**Affiliations:** ^1^Department of Assisted Reproductive Medicine, Shanghai First Maternity and Infant Hospital, Tongji University School of Medicine, Shanghai, China; ^2^Department of Assisted Reproduction, Shanghai Ninth Peoples Hospital, Shanghai Jiaotong University School of Medicine, Shanghai, China

**Keywords:** premature LH surge, progesterone protocol, late-follicular-phase ovarian stimulation, late-stimulation protocol, frozen-thawed embryo transfer

## Abstract

**Introduction:** A gonadotropin-releasing hormone antagonist is the most common modulator used to prevent the premature luteinizing hormone (LH) surge when ovarian stimulation was initiated in the late follicular phase. We aimed in this study to evaluate the feasibility of performing ovarian stimulation in the late follicular phase without the use of exogenous pituitary modulators.

**Methods:** Data were retrospectively collected from 404 normo-ovulatory patients who underwent their first *in vitro* fertilization (IVF)/intracytoplasmic sperm injection (ICSI) treatment in our department. One hundred sixteen subjects in the study group received ovarian stimulation when a dominant follicular diameter of ≥ 10 mm was confirmed by transvaginal ultrasonography after menstrual cycle day 6, which entailed a daily injection of gonadotropin until the trigger day, while 288 subjects in the control group received ovarian stimulation in the early follicular phase under a progesterone protocol. The primary outcome was the number of mature oocytes.

**Results:** There was no statistical difference in the number of mature oocytes between the two groups (9.67 ± 5.33 in the study group vs. 9.38 ± 5.15 in the control group, *P* = 0.693). No secondary LH surges in the study group and no premature LH surges in the control group were found during ovarian stimulation. The good-quality embryo rate per oocyte retrieved showed no significant difference between the two groups (35.22 vs. 35.91%, *P* = 0.665). The clinical pregnancy rate per transfer was 54.55% in the study group and 56.48% in the control group (*P* = 0.718), and the implantation rate was similar between the two groups (36.94 vs. 37.77%, *P* = 0.829).

**Conclusions:** Our study revealed that late follicular phase ovarian stimulation could be performed without an exogenous pituitary modulator.

## Introduction

Fresh-embryo transfer was once routine practice in the years before *in vitro* fertilization (IVF); however, its disadvantages included a high risk of ovarian hyperstimulation syndrome (OHSS) and the potentially detrimental effects of supraphysiologic hormone levels on endometrial receptivity. These adverse consequences have inspired experts to explore other strategies for improving the safety and outcomes of assisted reproductive treatments (1). Improvements in freezing/warming techniques have enabled thawed embryos to be preserved with an excellent survival rate. A recent large, multicenter, randomized trial involving 1,508 infertile women with polycystic ovary syndrome (PCOS) conducted by Chinese researchers demonstrated that frozen-embryo transfer (FET) was associated with a higher rate of live births and a lower risk of OHSS relative to fresh-embryo transfer (2). The reduction in the risk of OHSS with FET has also been confirmed among ovulatory women with infertility, although investigators have not found a statistical difference in the live-birth rate between FET and fresh-embryo transfers (3). In addition, this same team of researchers demonstrated that ovulatory women with a good prognosis and who received frozen, single-blastocyst transfer had a significantly higher rate of singleton live births than women receiving fresh, single-blastocyst transfer (4). In addition, a series of studies have shown that neonatal outcomes are improved in FET cycles compared with those from fresh IVF cycles (5). As a result, the proportion of FET cycles has consistently increased in many countries.

In combination with FET, physicians have conceived new ovarian stimulation protocols without needing to consider the potentially negative impacts that exogenous drugs or high hormonal concentrations imposed on the endometrium. Our present team of researchers was the first to introduce the use of exogenous progestational agents as an alternative to pituitary modulation by gonadotropin-releasing hormone agonists (GnRH-a) or GnRH antagonists for the prevention of premature LH surges in ovarian stimulation (6). Studies have been published on the comparison of progestins with GnRH analogs, and the usage of different types and dosages of progestins in various populations (7–12), and the investigators have affirmed that the oral administration of progestin from the early follicular phase (i.e., the “progesterone protocol”) was feasible in the regulation of LH levels, with reasonable pregnancy and neonatal outcomes (6–12). Meanwhile, with growing evidence supporting the theory of human ovarian follicular waves (13), a flexible method of ovarian stimulation has been proposed in which treatment may be initiated in the late follicular or luteal phase (14). This approach was initially employed in cancer patients for fertility preservation, with limited pregnancies following subsequent FET (14). At our center, we provide random-start ovarian stimulation protocols as an option for infertile patients who choose FET (15, 16).

GnRH antagonists constitute the most commonly used pituitary modulator when ovarian stimulation was initiated in the late follicular phase (14). We recently documented the initiation of ovarian stimulation with a dominant follicle diameter of ≥ 14 mm before spontaneous ovulation could be performed without exogenous pituitary modulators (17). Subsequently, we extended this method to patients who intended to undergo ovarian stimulation when the diameter of the dominant follicle was larger than 10 mm. Herein, we retrospectively screened the data from normo-ovulatory patients who underwent ovarian stimulation in the late-follicular phase with a dominant follicle diameter of ≥ 10 mm in the absence of exogenous pituitary modulators [i.e., a late-stimulation [LS] protocol], and compared the reproductive results with ovarian stimulation initiated in the early-follicular phase using a progesterone protocol.

## Materials and Methods

### Study Setting and Ethical Approval

This study was conducted at the Department of Assisted Reproduction of the Ninth People's Hospital of Shanghai Jiao Tong University School of Medicine (Shanghai, People's Republic of China), with the approval of the Institutional Review Board (IRB) of the Shanghai Ninth People's Hospital. All of the patients and their spouses provided informed consent with regard to their infertility treatments with IVF/ICSI procedures, and the use of medical records for research purposes was based on the principles of anonymity and confidentiality.

### Study Design and Population

This is a retrospective study in which we illustrated the clinical outcomes between ovarian stimulation using an LS protocol in normo-ovulatory patients and using a progesterone protocol. Our electronic medical record system did not contain the medical data of patients who started ovarian stimulation without oocytes retrieval, and the original data of unfinished cycles were not routinely kept; therefore, only women who underwent IVF/ICSI for the first time and completed oocytes retrieval with the “freeze-all” strategy from May 2016 to December 2018 were screened. We then followed up pregnancy results until April 2019. The inclusion criteria were as follows: (1) age < 40 years, (2) regular menstrual cycles in the range of 21–35 days over the past 6 months, (3) an antral follicle count (AFC) of >5 on menstrual cycle days (MC) 2–3, (4) a basal serum follicle-stimulating hormone (FSH) value < 10 IU/L, (5) a body mass index (BMI) of < 28 kg/m^2^, and (6) patients with a serum hormone determination and transvaginal ultrasonographic examination on MC 2–3. Patients who were missing core data or exhibited previous medical records, indicating poor ovarian reserve (such as elevated basal FSH ≥ 10 IU/L or AFC < 5 by ultrasonographic examination), were excluded from our study.

### Ovarian Stimulation Protocol and FET

The study group was treated using an LS protocol as follows: when the dominant follicular diameter of ≥ 10 mm was confirmed by transvaginal ultrasonography after MC6, we began ovarian stimulation with a daily injection of gonadotropin (Gn) until the trigger day. In the control group, 100 mg of a micronized progesterone soft capsule (brand name Utrogestan; Laboratories Besins International, France) was administered daily concomitant with Gn starting on MC 3 until the trigger day. Human menopausal gonadotropin (hMG) (brand name, fengyuan; Anhui Fengyuan Pharmaceutical Co., China) was the commonly used Gn in our clinic, since the price of hMG is only 1/7 of the price of urinary follicle-stimulating hormone (u-FSH) (brand name, lishenbao; Lizhu Pharmaceutical Trading Co., China) in China. The initiation dose of hMG (150–225 IU) was similar for both groups and could be adjusted thereafter, according to the size and number of developing follicles and serum concentrations of follicle-stimulating hormone (FSH), LH, estradiol (E_2_), and progesterone (P). If more than three dominant follicles reached 18 mm in diameter, oocyte maturation was triggered by 0.1 mg GnRH-a (Decapeptyl® Ferring Pharmaceuticals, Germany). We performed oocyte retrieval 36–38 h after the trigger using transvaginal ultrasound-guided follicle aspiration, and punctured all of the follicles larger than 10 mm. Then we performed *in vitro* fertilization, embryonic assessment, and vitrification in the usual manner as reported earlier (6–9). In brief, laboratory technicians carried out the *in vitro* fertilization by either conventional insemination or ICSI, depending upon semen parameters. Embryos underwent continuous examination, and good-quality embryos and blastocysts were frozen by vitrification.

Endometrial preparation before FET was done according to the standard protocols at our center (6–9), including natural cycle, stimulation cycle, and hormone-replacement therapy, based on the physicians' preference. Embryo transfer was performed on the third day after ovulation or progesterone administration, and blastocyst transfer was scheduled 5 days later. Luteal phase support was maintained until 10 weeks of gestation if the patient became pregnant.

### Outcome Measures

The primary outcome measure was the number of mature oocytes. Secondary outcome measures included the incidence of a secondary LH surge in the study group and a premature LH surge in the control group, the good-quality embryo rate per oocytes retrieved [percentage of good-quality Day 3 (D3) embryos and blastocysts divided by the number of retrieved oocytes], and the clinical pregnancy and implantation rates following FET.

### Statistical Analysis

Data are presented as means ± standard deviation (SD) for continuous variables, and as numbers and percentages for categorical variables. When continuous variables were normally or near-normally distributed, we used a Student's *t*-test; otherwise, a Mann–Whitney U test was used for non-normally distributed data. The chi-square test or Fischer's exact-probability test was adopted for categorical comparisons. Statistical significance was defined as *P* < 0.05. Data were analyzed using the Statistical Package for the Social Sciences for Windows, version 24.0 (SPSS, Chicago, IL, USA).

## Results

### Patient Characteristics

**Figure 1:**
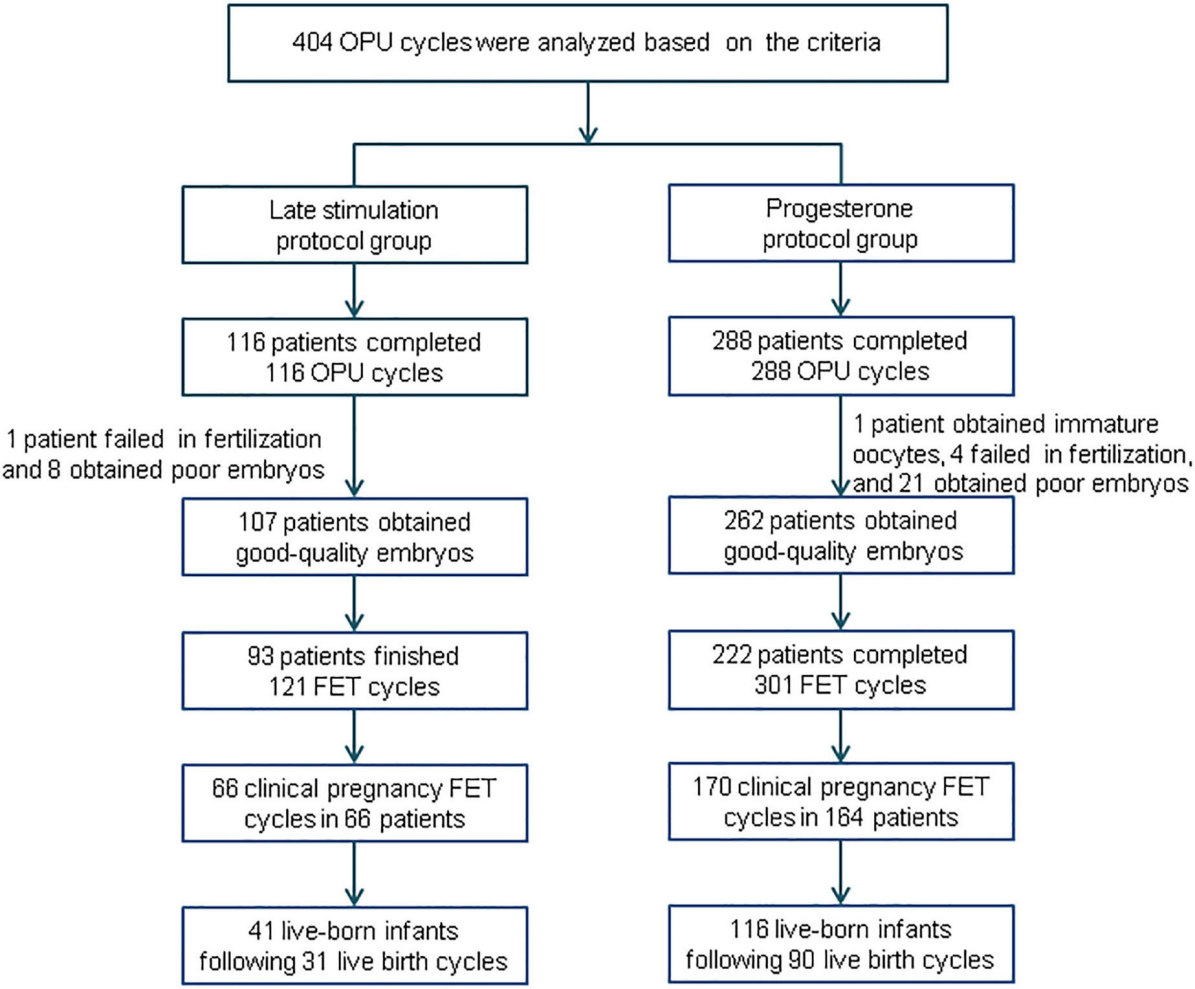
Flowchart of this study. OPU, oocyte pick-up; FET, frozen-thawed embryo transfer.

A summary profile of our study is presented in Figure 1. A total of 404 women were eligible to be analyzed: we enrolled 116 subjects in the study group and 288 subjects in the control group. After oocyte retrieval, 9 (7.76%) subjects in the study group did not generate a viable embryo, compared with 26 (9.03%) in the control group (*P* = 0.19). In the follow-up period, 54 patients did not complete their embryo transfers: 14 in the study group and 40 in the control group.

Basic characteristics of the patients in our study are depicted in Table 1. We uncovered no statistical differences between the two groups in age, BMI, duration of infertility, or basal serum hormone levels (basal FSH, LH, E_2_, and P). The proportion of primary or secondary infertility was comparable in the two groups.

**Table 1:** General information regarding patients undergoing IVF/ICSI treatment.

**Characteristic**	**Late stimulation group** **(*n =* 116)**	**Progesterone group** **(*n =* 288)**	***P*-value**
Age (years)	31.85 (3.08)	31.85 (3.67)	0.921
BMI (kg/m^2^)	20.69 (1.8)	20.9 (1.69)	0.159
Duration of infertility (years)	3.31 (2.5)	3.25 (2.28)	0.855
Type of infertility n (%)			0.198
Primary	69 (59.5)	151 (52.43)	
Secondary	47 (40.5)	137 (47.57)	
Antral follicle counts (n)	10.97 (4.8)	11.77 (6.53)	0.977
Indication for IVF/ICSI treatments (n)			0.574
Tubal factor	75	172	
Male factor	11	41	
Unknown factor	11	24	
Combined factors	19	51	
Basal FSH (IU/L)	5.47 (1.15)	5.47 (1.25)	0.979
Basal LH (IU/L)	3.62 (1.63)	3.88 (2.26)	0.686
Basal E_2_ (pg/mL)	36.23 (16.95)	36.03 (18.5)	0.658
Basal P (ng/mL)	0.31 (0.12)	0.3 (0.22)	0.1

### Ovarian Stimulation Characteristics and Embryologic Outcomes

Table 2 shows ovarian stimulation characteristics and embryologic outcomes in both the groups. Total hMG dose (1962.28 ± 517.06 vs. 1626.04 ± 311.75 IU) was greater, and the mean hMG duration (10.48 ± 2.44 vs. 8.78 ± 1.53) was longer in the study group relative to the control group, respectively (*P* < 0.001). The number of follicles with a diameter > 14 mm was similar between the groups, while the number of follicles with a diameter > 10 mm in the study group was greater than that in the control group (13.33 ± 6.67 vs. 11.87 ± 6.02, respectively; *P* = 0.02). The number of mature oocytes was not different: 9.67 ± 5.33 in the study group and 9.38 ± 5.15 in the control group (*P* = 0.693). The two groups were also comparable regarding the numbers of oocytes retrieved, fertilized oocytes, cleaved embryos, and good-quality embryos. Additionally, good-quality embryo rate per oocyte retrieved showed no significant difference between the two groups.

**Table 2:** Ovarian stimulation characteristics and embryo results of controlled ovarian stimulation in the two regimens.

**Characteristics**	**Late stimulation group** **(*n =* 116)**	**Progesterone group** **(*n =* 288)**	***P*-value**
hMG duration (days)	10.48 (2.44)	8.78 (1.53)	<0.001
hMG dose (IU)	1962.28 (517.06)	1626.04 (311.75)	<0.001
FSH on trigger day (IU/L)	14.37 (3.43)	13.58 (4.38)	0.223
LH on trigger day (IU/L)	1.8 (1.7)	2.62 (1.85)	<0.001
E2 value trigger day (pg/mL)	3496.93 (1464.45)	2963.6 (1722.69)	<0.001
*P*-value on trigger day (ng/mL)	8.67 (5.63)	4.43 (2.66)	<0.001
No. of > 10 mm follicles on trigger day	13.33 (6.67)	11.87 (6.02)	0.02
No. of > 14 mm follicles on trigger day	10.98 (6.21)	10.23 (5.84)	0.184
No. of oocytes retrieved	10.99 (5.88)	10.75 (5.87)	0.707
No. of MII oocytes	9.67 (5.33)	9.38 (5.15)	0.693
No. of fertilized oocytes (2PN)	7.61 (4.7)	7.13 (4.35)	0.431
No. of cleaved embryos	7.49 (4.65)	7 (4.27)	0.424
No. of Day 3/4 viable embryos	2.97 (2.27)	3.01 (2.19)	0.785
No. of Day 5/6/7 viable embryos	0.9 (1.17)	0.85 (1.25)	0.563
No. of all the viable embryos	3.87 (2.78)	3.86 (2.77)	0.968
Oocyte retrieval rate (%)	69.75% (1,275/1,828)	69.53% (3,097/4,454)	0.866
Mature oocyte rate (%)	88% (1,122/1,275)	87.21% (2,701/3,097)	0.476
Fertilization rate (%)	78.7% (883/1,122)	76.01% (2,053/2,701)	0.073
Cleavage rate (%)	98.41% (869/883)	98.25% (2,017/2,053)	0.747
Viable embryos per oocyte retrieved (%)	35.22% (449/1,275)	35.91% (1,112/3,097)	0.665

*For categorical variables, % (n) is presented; for continuous variables, the mean (SD) is presented; for comparison between groups, Pearson's chi-square test or Fisher's exact test was used for categorical variables, and the Mann–Whitney U-test was used for continuous variables*.

### Pregnancy Outcomes Following Frozen-Thawed Embryo Transfer

In this study, 315 women completed a total of 422 FET cycles; including 70 women who finished two FET cycles, 11 women who finished three FET cycles, and 5 women who finished four FET cycles (Table 3). A total of 786 embryos were thawed, and all of the frozen embryos survived. In the study group, the numbers of FET cycles with one D3 embryo, two D3 embryos, one blastocyst, and two blastocysts were 11, 97, 10, and 3, respectively. In the P group, one Day 3 embryo was transferred in 17 cycles, two D3 embryos were transferred in 251 cycles, one blastocyst was transferred in 21 cycles, and two blastocysts were transferred in 12 cycles. The method of endometrial preparation was comparable between the two groups.

**Table 3:** Pregnancy and live-birth outcomes of frozen-thawed embryos derived from the two regimens.

**Outcomes**	**Late stimulation group**	**Progesterone group**	***P*-value**
No. of patients (n)	93	222	0.523
With 1 FET cycle	68	161	
With 2 FET cycles	22	48	
With 3 FET cycles	3	8	
With 4 FET cycles	0	5	
No. of thawed embryos (n)	1.82 ± 0.39	1.87 ± 0.33	0.293
No. of FET cycles (n)	121	301	0.494
With 1 day-3 embryo transferred	11	17	
With 2 day-3 embryos transferred	97	251	
With 1 blastocyst transferred	10	21	
With 2 blastocysts transferred	3	12	
Endometrium preparation (n)			0.27
Natural cycle	67	143	
Mild stimulation	47	143	
HRT	7	15	
**Pregnancy outcome of FET % (n)**			
Biochemical pregnancy rate per transfer	61.16% (74/121)	61.79% (186/301)	0.903
Clinical pregnancy rate per transfer	54.55% (66/121)	56.48% (170/301)	0.718
Clinical pregnancy per patient	70.97% (66/93)	76.58% (170/222)	0.295
Multiple pregnancy rate	24.24% (16/66)	27.65% (47/170)	0.596
Implantation rate	36.94% (82/222)	37.77% (213/564)	0.829
Ectopic pregnancy rate	1.52% (1/66)	2.94% (5/170)	0.532
Intrauterine and ectopic pregnancy rate	0% (0/66)	0.59% (1/170)	0.532
Early miscarriage rate	4.55% (3/66)	8.24% (14/170)	0.325
**Live-birth outcomes**			
Live birth cycle (n)			0.834
Term delivery	26	74	
Preterm delivery	5	16	
Newborn			0.662
Single birth (n)	21	64	
Twin birth (n)	20	52	
Single birthweight (g)	3310.95 ± 353.45	3246.72 ± 476.31	0.572
Twin birthweight (g)	2480.5 ± 485.54	2468.08 ± 446.32	0.942

*For categorical variables, % (n) is presented; for continuous variables, the mean (SD) is presented; for comparison between groups, Pearson's chi-square test or Fisher's exact test was used for categorical variables, and the Mann–Whitney U-test was used for continuous variables. FET, frozen embryo transfer*.

The clinical pregnancy rate per transfer was 54.55% (66/121) in the study group and 56.48% (170/301) in the control group (*P* > 0.05), while the implantation rate was similar between the two groups (36.94 vs. 37.77%, respectively; *P* = 0.829). We found no differences in the rate of biochemical pregnancy, multiple pregnancy, ectopic pregnancy, or early miscarriage (*P* > 0.05). One hundred and twenty-one patients completed delivery during our follow-up, including 26 patients with term delivery in the study group and 74 patients with term delivery in the control group (*P* > 0.05). Birth weights of the singleton newborns and twin newborns showed no significant difference between the two groups.

### Dynamic Changes in Hormonal Levels During Ovarian Stimulation

**Figure 2:**
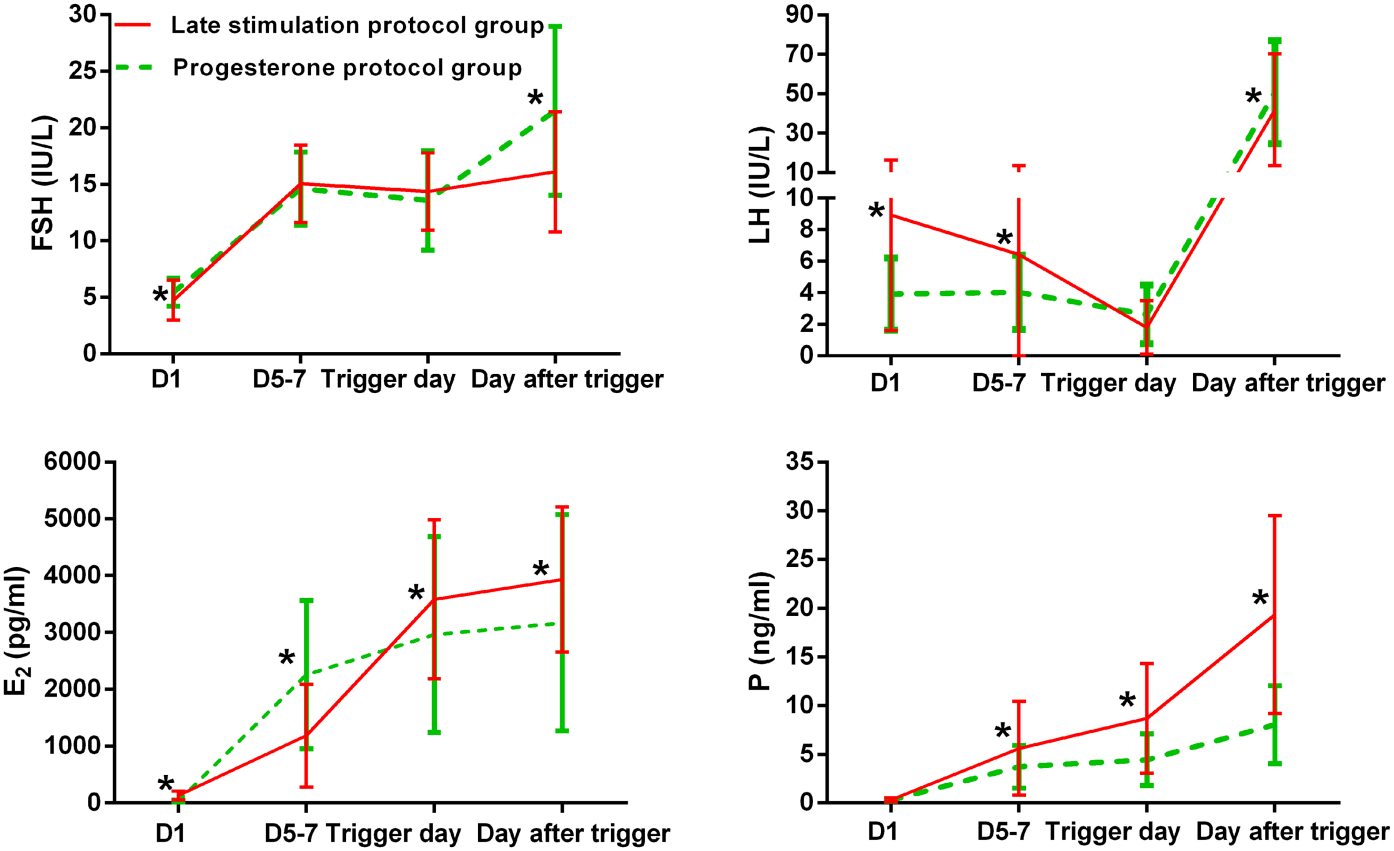
Serum hormonal profiles during ovarian stimulation in the two groups. The green lines represent the control group (progesterone protocol group), and the red lines represent the study group (late stimulation protocol group). *Time point at which *P* < 0.05. The initial day of ovarian stimulation is denoted as day 1 (D1). FSH, follicle-stimulating hormone; LH, luteinizing hormone; E_2_, estradiol; P, progesterone.

Figure 2 shows serum hormone concentrations of FSH, LH, E_2_, and P in the two groups, with the starting day denoted as day 1 (D1). None of the patients in the study group experienced secondary premature LH surges, only the spontaneous ovulatory LH surge of the leading follicle. In the study group, the average E_2_ on D 5–7 was lower than in the control group, and later increased above levels in the control group on the day after the trigger (*P* < 0.05). Serum P in the study group was significantly higher than in the control group on D 5–7, trigger day, and the day after the trigger (*P* < 0.05).

We did not observe ovulation in six patients of the study group who exhibited a range of serum P levels from 0.5 to 1 ng/mL, and the variations in serum LH levels were below 5 IU/L. These six patients produced one to eight frozen embryos, of which three patients delivered successfully, two have ongoing pregnancies, and one had an early miscarriage.

## Discussion

In this retrospective cohort study, we compared the hormonal dynamics, embryonic parameters, and pregnancy outcomes following FET cycles in normo-ovulatory patients using an LS protocol vs. progesterone protocol. No secondary LH surges in the study group and no premature LH surges in the control group were found during ovarian stimulation, and the number of mature oocytes was comparable between the two groups. We also did not observe any statistical differences in the clinical pregnancy rate or implantation rate. Our results suggest that ovarian stimulation initiated in the late follicular phase can be performed in the absence of exogenous pituitary modulators without compromising the quality of oocytes/embryos.

In our study, the dominant follicle ovulated before the majority of the secondary follicles surpassed 10 mm in a large proportion of patients using the LS protocol. These patients did not require the addition of exogenous GnRH antagonists or progestin for LH suppression because the early-onset LH peaks induced by the preovulatory follicles growing from the initial subordinate follicles could be suppressed by endogenous P secretion following ovulation of the dominant follicle (17). However, if most of the subordinate follicles surpass 10 mm before the spontaneous LH surge, the risk of a premature LH surge will increase without exogenous pituitary modulators. In such cases, Cakmak et al. (14) recommended the addition of GnRH antagonists from the beginning of ovarian stimulation until the day of the trigger, and Qin et al. (16) administered exogenous progestin throughout the process of ovarian stimulation to prevent premature LH surges. There were six patients whose subordinate follicles developed to > 10 mm before the spontaneous LH surge in the study group, but we did not observe ovulation in these patients throughout ovarian stimulation, and no premature LH surges were detected in these patients. In our prior study, there were 4 of 70 patients who underwent the LS protocol whose dominant follicles failed to ovulate (17). These cases indicated that the LH regulatory mechanism varies widely by individuals, and that it may be superfluous to add exogenous pituitary modulators even if ovarian stimulation commenced in the late follicular phase. The discrepancy in LH secretion in different populations raises the question of how to determine whether a pituitary modulator should be administered or not; this is currently undetermined and requires further exploration.

Conventional wisdom dictates that GnRH antagonists can rapidly attenuate circulating concentrations of serum LH by competitively blocking pituitary GnRH receptors. When GnRH antagonists were first introduced for the prevention of a premature LH surge, they were administered from the beginning of ovarian stimulation until the trigger day, a “fixed protocol” as we now refer to it. Subsequently, the flexible GnRH antagonist protocol was proposed to reduce the number of injections and, thus, reduce the economic burden to a patient. The addition of GnRH antagonist was initially primarily based on the duration of Gn stimulation or the diameter of the follicles present. However, Liu et al. (18) reported that patients with sustained, low levels of LH (with a maximal LH level <4 IU/L) might not require GnRH antagonist addition during ovarian stimulation using a flexible GnRH antagonist protocol; this is commensurate with our results. The LH levels were below 5 IU/L throughout the process of ovarian stimulation in our six patients without ovulation of the initial dominant follicle in the study group. Moreover, the aforementioned authors observed a detrimental effect of excessive LH suppression on reproductive outcomes (17). As reported in earlier studies, the concept of an “LH window” has been proposed for decades, which posits that there is an optimal range in serum LH levels for adequate follicle development, from 1.2 to 5.0 IU/L (19). From this perspective, the unnecessary administration of exogenous pituitary modulators for patients with low LH levels may result in a further diminution in serum LH concentrations, which will adversely affect clinical results.

Despite the lack of relevant published articles, tailoring the dose of Gn, and exogenous pituitary modulators depends upon the number and diameter of developing follicles, as well as serum hormone concentrations, and is the usual practice for our department. Based on our clinical experience, the response of the pituitary to endogenous and exogenous progesterone is complicated, which is different from the dose-dependent effects of GnRH antagonist. As reported in a dose-determining investigation of progesterone protocols with Utrogestan (a type of progesterone soft capsule), we found that the dose of Utrogestan was not associated with circulating serum LH concentrations (6). One possible reason for this is that the absorption of progestins differs from person to person (6); in addition, E_2_ might play a synergistic role in LH suppression, which may constitute another aspect that contributes to additional complications in the regulation of LH secretion by progesterone (6). Progestin administration in the method of flexible GnRH antagonist protocol failed to induce a rapid LH suppression in some patients (unpublished data). Nevertheless, researchers in Japan recently reported the effectiveness of a progestin when started later in ovarian stimulation during the early follicular phase using a flexible GnRH antagonist protocol (11), as they detected no premature ovulations in their study (11). We hypothesize that this discrepancy is due to individual differences in ovarian reserve. In the retrospective self-case-control study by the Japanese investigators, the patients were oocyte donors 20–35 years of age. Unfortunately, the authors did not measure serum LH levels during the ovarian stimulation, a major shortcoming. According to our clinical observations, ovarian reserve was evidently diminished in patients whose elevated LH levels failed to be promptly suppressed by exogenous progestin in the flexible method (unpublished data). This corresponded with a prior case–control study where the researchers explored risk factors for breakthrough LH surge in a flexible GnRH antagonist protocol (20). In their study, 37 (0.34%) of the 10,809 antagonist cycles encountered breakthrough LH surges during a 9-year study period (20), and the patients with breakthrough LH surges were significantly older, with higher basic FSH levels and lower numbers of antral follicle. The authors thus recommended doubling the dose of GnRH antagonist in subsequent cycles for patients with a history of breakthrough ovulation, despite GnRH antagonist administration (20). Though it is a rare event that the usage of GnRH-a, GnRH-ant, or progesterone fails to suppress the pituitary, we recommend that when using an exogenous pituitary modulator, one should take into account the fluctuation of serum LH levels, the ovarian reserve, and history of ovarian stimulation for a better management of ovarian stimulation by individuals.

The dose and duration of hMG administration were higher in the study group relative to the control group, a finding that is consistent with several previous studies using a randomly started approach to ovarian stimulation (14, 16). The mean E_2_ and P levels during ovarian stimulation were also different between the two groups, which may result in a discrepancy in the extent of LH suppression, and then affect ovarian sensitivity to Gn. Nevertheless, whether there is a cutoff level of serum P concentration above which ovarian suppression is observed remains to be seen. Despite the higher total dose of Gn used, the expense of exogenous modulators is abolished. Medico-economic analyses are still needed in further prospective studies to demonstrate a beneficial effect of this novel protocol that will lower overall cost, while still considering expenditures for FET.

The pattern of follicular development in late-follicular-phase ovarian stimulation was similar to the asynchronized follicular development observed in ovarian stimulation and remains a great challenge to understand. From our perspective, ovarian stimulation in the late follicular phase provides us a novel perspective from which to assess the asynchronous follicular development during ovarian stimulation. Baerwald and co-workers demonstrated that the number of dominant follicles could be increased by synchronizing ovarian stimulation with follicle recruitment/wave emergence in women using a GnRH antagonist protocol and who exhibited a previous suboptimal follicular response (21). Based on the theory of follicular waves, the day prior to ovulation was considered to be the time of wave emergence (13). For our patients in the study group with ovulation of the dominant follicle, exogenous Gn administration on the day before wave emergence could theoretically increase the number of dominant follicles by prolonging the FSH “gate,” above which antral follicles would be selected to grow. This postulate was consistent with our results, which showed that the number of dominant follicles was significantly greater in the study group compared with the control group. Additionally, some researchers hold the opinion that the functional ovarian cysts following GnRH-a administration during ovarian stimulation provide a negative influence on follicular recruitment by reducing the space for follicle growth and the ovarian blood supply for subordinate follicles (22). However, suppressive effects of the unruptured dominant follicle on the development of subordinate cohorts were not observed in our study. Further assessments on a larger scale are still needed to determine whether the unruptured follicle during ovarian stimulation using the LS protocol exerts any detrimental impacts on ovarian response and oocyte competence.

The limited number of cases and retrospective design of our study resulted in some bias. Continuous follow-up is also required to determine the long-term safety of children conceived using this novel protocol. Besides, owing to the fact that the data of patients who started ovarian stimulation without egg collection are not available, only ovarian stimulation cycles with oocyte retrieval were analyzed in our study, and further prospective studies including all the initiated ovarian stimulation cycles are therefore needed to evaluate the efficiency of late follicular phase ovarian stimulation without exogenous pituitary modulators in a larger sample size.

## Conclusions

In this study, we demonstrated that ovarian stimulation in the late follicular phase could be performed without exogenous pituitary modulators, which provides an effective alternative for patients who choose FET during infertility treatments or fertility preservation.

## Data Availability Statement

All datasets generated for this study are included in the article/supplementary material.

## Ethics Statement

The studies involving human participants were reviewed and approved by Institutional Review Board (IRB) of Shanghai Ninth People's Hospital. All of the patients and their spouses provided informed consent with regard to their infertility treatments with IVF/ICSI procedures, and written informed consent for the use of medical records for research purposes was not required according to the national legislation and the institutional requirements.

## Author Contributions

XZ and JY collected and analyzed the data. XZ wrote the manuscript. YF conceived the protocol and supervised the study. All authors contributed to the article and approved the submitted version.

## Conflict of Interest

The authors declare that the research was conducted in the absence of any commercial or financial relationships that could be construed as a potential conflict of interest.
